# *Aureobasidium pullulans* produced β-glucan is effective to enhance Kurosengoku soybean extract induced Thrombospondin-1 expression

**DOI:** 10.1038/s41598-017-03053-9

**Published:** 2017-06-06

**Authors:** Daisuke Muramatsu, Mitsuyasu Okabe, Akinori Takaoka, Hiroshi Kida, Atsushi Iwai

**Affiliations:** 1 Aureo Science Co., Ltd., Hokudai Business Spring, North 21, West 12, Kita-ku, Sapporo, Hokkaido 001-0021 Japan; 2 Aureo Co., Ltd., 54-1 Kazusakoito, Kimitsu, Chiba 292-1149 Japan; 3 Division of Signaling in Cancer and Immunology, Institute for Genetic Medicine, Hokkaido University, North 15, West 7, Kita-ku, Sapporo, Hokkaido 060-0815 Japan; 4 Hokkaido University Research Center for Zoonosis Control, North 20, West 10, Kita-ku, Sapporo, Hokkaido 001-0020 Japan

## Abstract

Black yeast, *Aureobasidium pullulans* is extracellularly produced β-(1,3), (1,6)-D-glucan (β-glucan) under certain conditions. In this study, using *Glycine max* cv. Kurosengoku (Kurosengoku soybeans), the production of β-glucan through fermentation of *A*. *pullulans* was evaluated, and the effects of *A*. *pullulans* cultured fluid (AP-CF) containing β-glucan made with Kurosengoku soybeans (kAP-CF) on a human monocyte derived cell line, Mono Mac 6 cells were investigated. Concentration of β-glucan in kAP-CF reached the same level as normal AP-CF. An anti-angiogenic protein, Thrombospondin-1 (THBS1) was effectively induced after the stimulation with kAP-CF for comparison with AP-CF. The THBS1 is also induced after stimulation with hot water extract of Kurosengoku soybeans (KS-E), while the combined stimulation of β-glucan with KS-E more effectively induced THBS1 than that with KS-E alone. These results suggest effects of *A*. *pullulans*-produced β-glucan on the enhancement of Kurosengoku soybean-induced THBS1 expression.

## Introduction

A black yeast, *Aureobasidium pullulans*, extracellularly produces a β-(1, 3), (1, 6)-D-glucan (β-glucan) under certain conditions. The *A*. *pullulans*-produced β-glucan is soluble, and consists of a β-(1, 3)-linked glucose main chain and β-(1, 6)-linked glucose branches^1, 2^. The *A*. *pullulans*-produced β-glucan is known to be a dietary fiber and also an immune stimulator, and it is believed to provide beneficial effects to health through its function as a dietary fiber and its immuno-stimulating activity. Actually, anti-tumor^3–5^, anti-infectious diseases^6, 7^, anti-non nonalcoholic fatty liver disease (NAFLD)^8^, and anti-atherosclerosis^9^ effects of *A*. *pullulans*-produced β-glucan under experimental conditions have been reported.

Soybeans (*Glycine max* (L.) Merrill) are considered a health promoting food, and beneficial effects including anti-tumor, anti-diabetic, and anti-obesity effects of soybean components, such as isoflavones^10–13^, saponin^10, 14^, lecithin^15, 16^, and soybean protein^17, 18^ on health have been reported. The black soybean, Kurosengoku (*Glycine max* cv. Kurosengoku) is a local variety cultivated in Hokuryu town, Hokkaido, Japan. Kurosengoku soybeans are smaller than other soybeans, have a thick husk, and are characterized as containing larger amounts of anthocyanins and lipids when compared with other soybean varieties. Anthocyanins are known to be antioxidative agents, and are effective to ameliorate oxidative stress-related diseases such as cardiovascular disease^19^. Inflammation induced oxidative stress is reduced by treatment with β-glucan produced by *A*. *pullulans* through its immuno modulating function^20^. However, any direct antioxidant activity of *A*. *pullulans*-produced β-glucan as an antioxidant agent is weak^21^. This suggests that by the combined administration of β-glucan-containing *A*. *pullulans*-cultured fluid (AP-CF) together with Kurosengoku soybeans, the effects on health would be complementarily improved through the antioxidant effect of the Kurosengoku soybeans. In addition, it has been reported that Kurosengoku soybeans activate type-1 immunity in a Toll-like receptor (TLR)4- and TLR2-dependent manner^22^. Activation of type-1 immunity is involved in the prevention of tumor^23^ formation and allergic diseases^24^. These effects resemble the effects of β-glucan, and may be anticipated to be enhanced by the combined administration of *A*. *pullulans* together with Kurorengoku soybeans.

In the present study, to utilize the beneficial effects of Kurosengoku soybeans AP-CF, the production of β-glucan through fermentation of *A*. *pullulans* using Kurosengoku soybeans was evaluated, and the effects of the AP-CF produced using Kurosengoku soybeans (kAP-CF) on culture cells were investigated. The results show that *A*. *pullulans* also effectively produces β-glucan with Kurosengoku soybeans as a nitrogen source with similar efficacy as with conventional methods using rice bran. The AP-CF prepared with Kurosengoku soybeans (kAP-CF) exhibited significantly higher Thrombospondin-1 (THBS1) induction activity in the human monocyte-derived cell line Mono Mac 6^25^, higher than with AP-CF prepared by conventional methods. The THBS1 is known to be an endogenous angiogenic inhibitor, and is involved in the inhibition of tumor angiogenesis and growth^26, 27^. Hot-water extracts of Kurosengoku soybeans (KS-E) have also indicated THBS1 induction activity, and the THBS1 induction after stimulation with KS-E was enhanced after co-stimulation with conventional AP-CF and purified β-glucans. The THBS1 induction activity of KS-E is mainly dependent on soy isoflavones, and the THBS1 induction activities of daidzein and genistein are higher than that of glycitein. These results suggest that β-glucan is effective in enhancing the effects of soy isoflavones on the cells.

## Results

### Stimulation with *Aureobasidium pullulans*-cultured fluid (AP-CF) made with Kurosengoku soybeans (kAP-CF) effectively induces Thrombospondin-1 (THBS1) in Mono Mac 6 cells

Initially, production of *A*. *pullulans*-fermented β-glucan using powdered Kurosengoku soybeans as the nitrogen source was investigated. Conventionally, *A*. *pullulans*-cultured fluid (AP-CF) containing β-glucan is prepared using rice bran as the nitrogen source. The concentration of β-glucan in AP-CF made with Kurosengoku soybeans as the nitrogen source (kAP-CF) was estimated to be 6 mg/ml, a level similar to conventional AP-CF produced with rice bran. The results indicate that the ground Kurosengoku soybeans may be substituted for and used as the nitrogen source in the fermentation of *A*. *pullulans* for β-glucan production.

**Figure 1:**
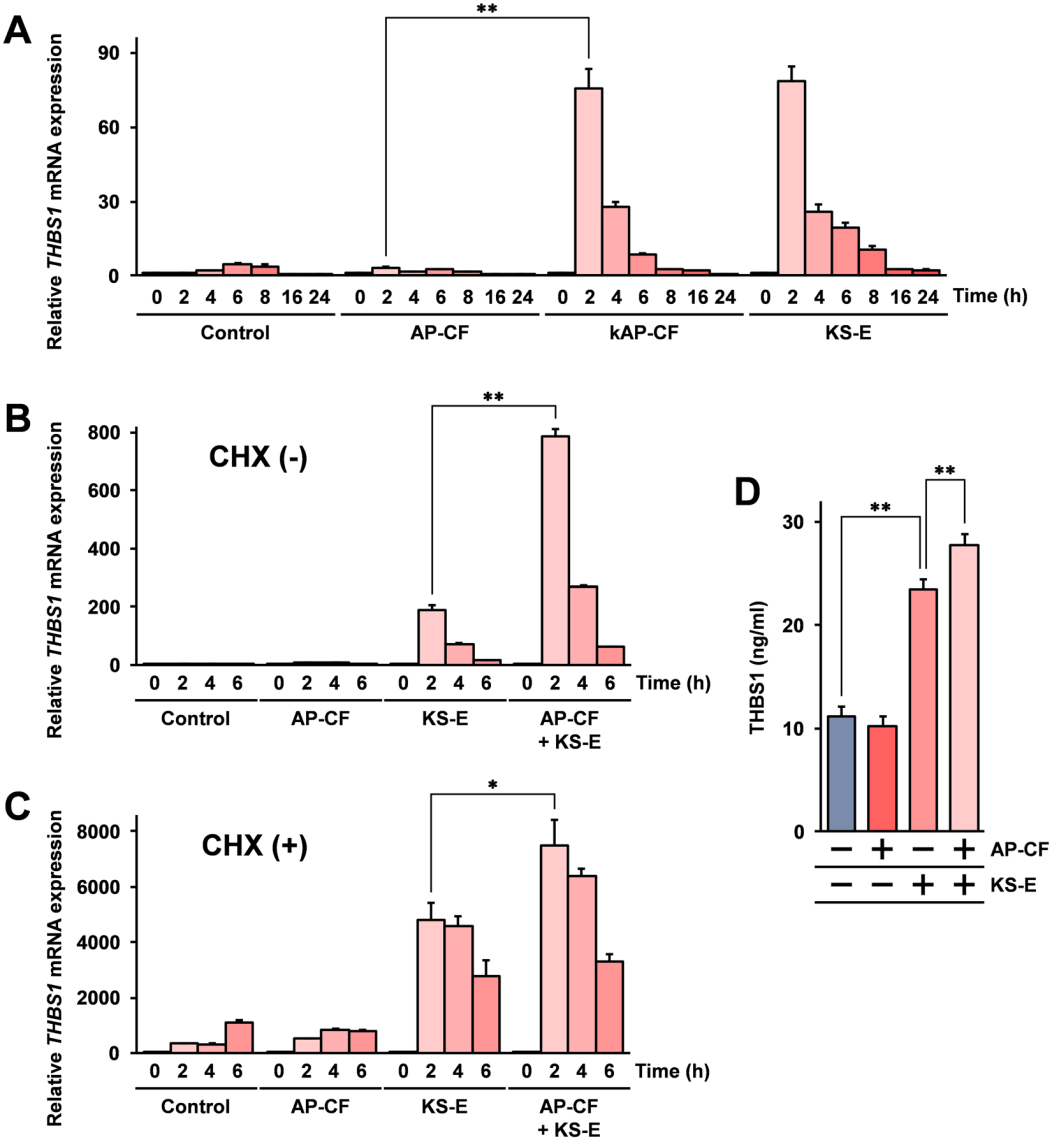
*Aureobasidium pullulans*-cultured fluid (AP-CF) enhances Thrombospondin-1 (THBS1) expression after stimulation with hot water extracts of Kurosengoku soybeans (KS-E). (**A**) Mono Mac 6 cells were stimulated with AP-CF or AP-CF made with ground powder of Kurosengoku soybeans (kAP-CF) as a nitrogen source, at the concentration of 100 μg/ml β-glucan, or the cells were stimulated with KS-E at 20-fold dilution. (**B**,**C**) Mono Mac 6 cells were treated with or without 5 mM cycloheximide (CHX) for 30 min. Subsequently, the cells were stimulated with KS-E at 20-fold dilution together with AP-CF at the concentration of 100 μg/ml β-glucan. After the incubation period indicated in the figure, the cells were harvested, and the THBS1 mRNA expressions were measured using the real-time RT-PCR method. The data are represented as relative values compared with the mRNA expression at the 0-hour time point after the normalization with glyceraldehyde 3-phosphate dehydrogenase (GAPDH) mRNA expression. Error bars indicate standard deviations calculated by three independent experiments. (**D**) Mono Mac 6 cells were stimulated with KS-E at 20-fold dilution together with AP-CF at the concentration of 100 μg/ml β-glucan. After 24 hours, concentrations of THBS1 in the supernatant of a cultured medium of the cells were measured by ELISA. Error bars indicate standard deviations calculated by three independent experiments. The asterisk (*) and double asterisks (**) indicate p < 0.05 and p < 0.01 respectively.

Next, to assess the kAP-CF and compare it with conventional AP-CF made with rice bran, a human, acute monocytic leukemia derived cell line, Mono Mac 6 cells were stimulated with kAP-CF, and the expression of mRNAs was evaluated using primer arrays. The primer array analysis showed that there is significant expression of Thrombospondin-1 (THBS1) mRNA after stimulation with kAP-CF. As shown in Fig. 1A, the THBS1 mRNA expression was statistically significantly increased after stimulation with kAP-CF, with only weak THBS1 mRNA induction in conventionally stimulated AP-CF cells; the stimulation with hot water extracts of Kurosengoku soybeans (KS-E) also effectively induced THBS1 mRNA.

The kAP-CF contained 0.3% (W/V) powdered Kurosengoku soybeans, while KS-E was extracted with 30% (W/V) of Kurosengoku soybeans. These results indicate that the stimulation with kAP-CF is more effective in inducing THBS1 mRNA than KS-E, suggesting that the β-glucan in the AP-CF may be involved in the enhancement of THBS1 mRNA expression after stimulation with KS-E, and next the effects of β-glucan containing AP-CF on the enhancement of THBS1 mRNA expression after stimulation with KS-E were investigated. The results show that conventional AP-CF made without Kurosengoku soybean addition enhances the KS-E induced THBS1 mRNA expression (Fig. 1B). The induction of THBS1 mRNA after stimulation with AP-CF and KS-E are transient and peaked at 2 hours after the stimulation, suggesting that the induction of THBS1 mRNA is a primary response to the simulation. To confirm this, the THBS1 mRNA expression was analyzed with protein synthesis inhibited by a high concentration of cycloheximide (5 mM). Under this condition, new protein synthesis is almost completely blocked^28^. As shown in Fig. 1C, THBS1 mRNA was effectively induced after stimulation with KS-E, and the induction of THBS1 mRNA was enhanced after the combined stimulation with AP-CF. The results indicate that the THBS1 mRNA induction after stimulation with KS-E and the co-stimulation effects of AP-CF on THBS1 mRNA induction are the primary responses of the cells to the stimulations, and that new protein synthesis is not required for the THBS1 mRNA induction.

To confirm the induction of THBS1 after stimulation with KS-E at the protein level, the amount of THBS1 protein in the supernatant of the culture medium was monitored by ELISA. The results show that the THBS1 protein increased significantly after stimulation with KS-E, and the induction of THBS1 protein was statistically significantly enhanced after co-stimulation with AP-CF (Fig. 1D). These results show that the stimulation with KS-E induces THBS1 in Mono Mac 6 cells, and that AP-CF is involved in enhancing the THBS1 induction.

### β-glucan is involved in the enhancement of the THBS1 induction after stimulation with KS-E

**Figure 2:**
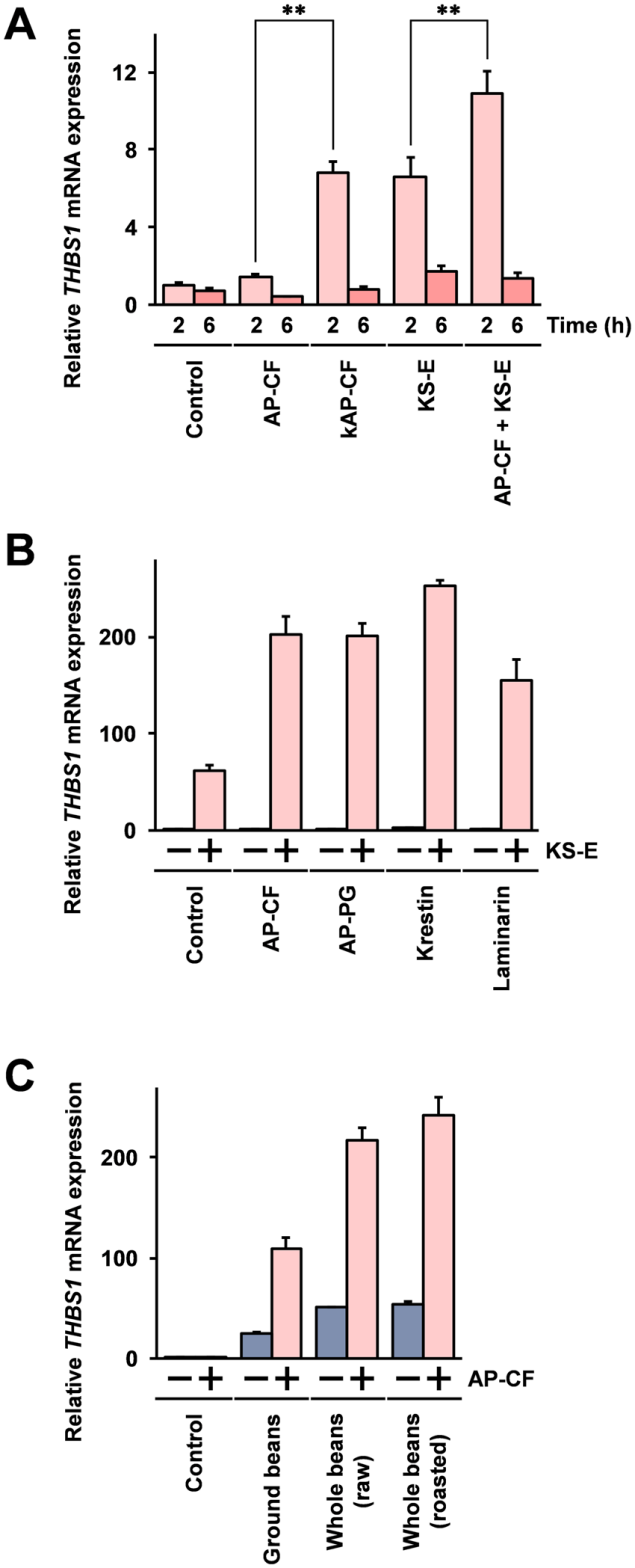
β-glucan is involved in the enhancement of THBS1 expression after stimulation with KS-E. (**A**) THP-1 cells were stimulated with conventional AP-CF or AP-CF made using ground powder of Kurosengoku soybeans (kAP-CF) as a nitrogen source, at the concentration of 100 μg/ml β-glucan, or the cells were stimulated with KS-E at 20-fold dilution. The cells were harvested at the time points indicated in the figure, and the THBS1 mRNA expressions in the cells were measured using the real-time RT-PCR method. (**B**) Mono Mac 6 cells were stimulated with AP-CF, AP-PG, Krestin, or laminarin at concentrations of 100 μg/ml β-glucan, with or without 20-fold dilution of KS-E. (**C**) Mono Mac cells were stimulated with a 20-fold dilution of KS-E from ground beans, whole raw beans, or whole roasted beans, with or without AP-CF at a concentration of 100 μg/ml β-glucan. After 2 hours, the cells were harvested, and then the total RNA prepared from the cells was subjected to real-time RT-PCR analysis. The data are represented as relative values compared with the mRNA expression in unstimulated control cells after normalization with the GAPDH mRNA expression. Error bars indicate standard deviations calculated by three independent experiments.

To assess the THBS1 induction identified in Mono Mac 6 cells after stimulation in other cell lines, a human monocyte derived cell line, THP-1 cells^29^ were used, and the responses to the stimulation with KS-E were investigated. As shown in Fig. 2A, stimulation with KS-E also effectively induces THBS1 mRNA expression in THP-1 cells, similar to that in the Mono Mac 6 cells. In addition, co-stimulation with AP-CF significantly enhanced THBS1 mRNA induction after the stimulation with KS-E.

β-glucan is the main component of AP-CF and thought to be the most important component providing most of the beneficial effects of AP-CF. To investigate whether the effects of AP-CF on the enhancement of the THBS1 induction after stimulation with KS-E depend on β-glucan, *A*. *pullulans*-produced purified β-glucan (AP-PG) was prepared from AP-CF, and the effects of β-glucan on KS-E induced THBS1 mRNA expression were examined. As shown in Fig. 2B, the results show that AP-CF and AP-PG, purified β-glucan from AP-CF, exhibit very similar activities after the enhancement by KS-E induced THBS1 mRNA. The results suggest that the enhancement of KS-E induced THBS1 expression depends on the β-glucan in AP-CF.

In addition to AP-PG, other commercially available β-glucans were also investigated. Krestin is a β-glucan derived from a mushroom, *Trametes versicolor*, and laminarin is a seaweed, *Laminaria digitata*, derived β-glucan. Like *A*. *pullulans* produced β-glucan, these β-glucans consist of β-1,3- and β-1,6-linked glucose residues, however all of these β-glucans are structurally distinct^2, 30, 31^. The results show that both Krestin and laminarin enhance the THBS1 mRNA induced response to the stimulation with KS-E.

Kurosengoku soybeans are also ingested as a tea using roasted beans, and the activity of KS-E prepared from roasted beans as it affects the THBS1 mRNA induction was investigated. As shown in Fig. 2C, KS-E prepared from the roasted beans also induced THBS1 mRNA statistically significantly after the stimulation. Here, stimulation with KS-E prepared from the whole beans induced THBS1 mRNA more strongly than with KS-E prepared from ground Kurosengoku soybean powder. These results suggest that components contained in the shell or germ of the Kurosengoku soybeans may be involved in the induction of THBS1.

### Isoflavones contained in Kurosengoku soybeans are involved in the induction of THBS1

**Figure 3:**
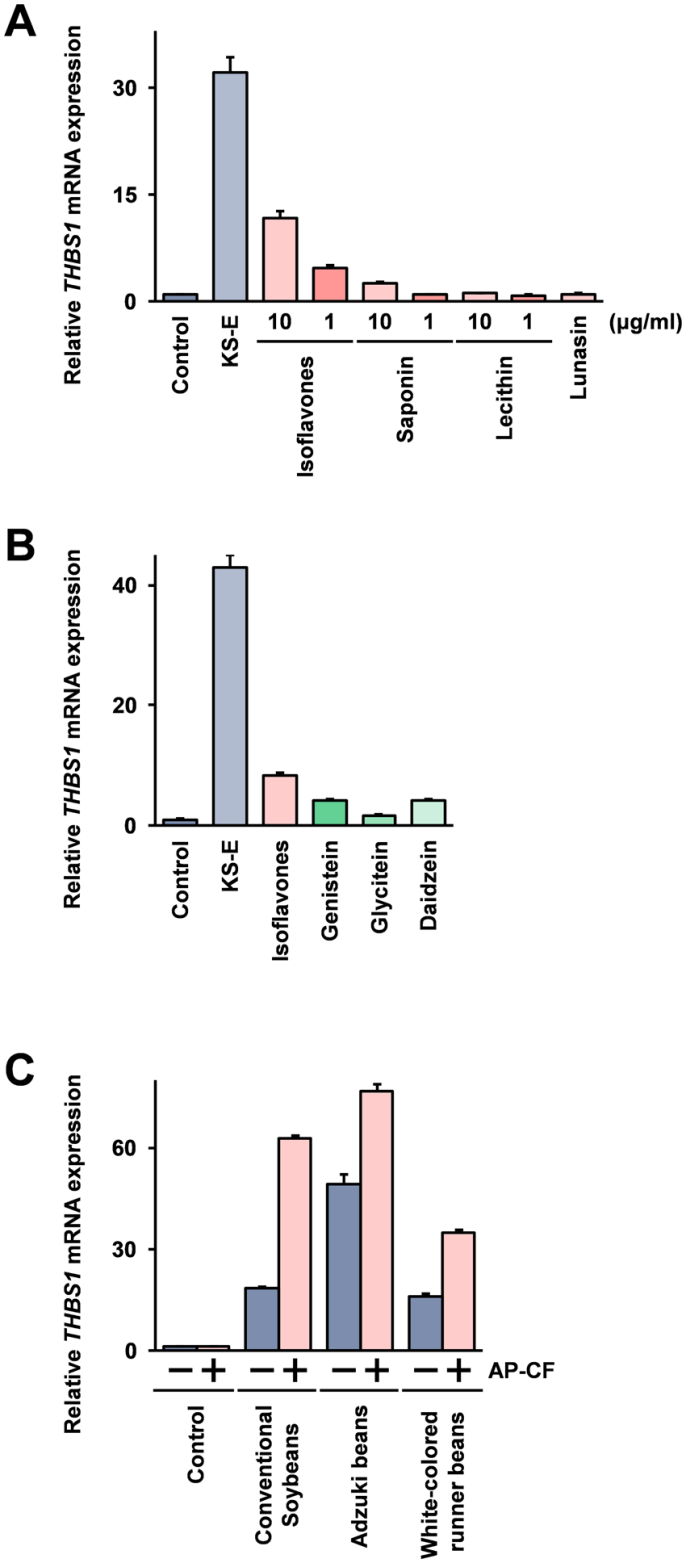
Soy isoflavones are involved in the induction of THBS1. (**A**) Mono Mac 6 cells were stimulated with a 20-fold dilution of KS-E, 20 μM Lunasin, or isoflavone mixture, saponin, and lecithin isolated from soybeans at the 1 or 10 μg/ml concentrations indicated in the Figure. (**B**) Mono Mac 6 cells were stimulated with a 20-fold dilution of KS-E, or 10 μg/ml isoflavone mixture of genistein, glycitein, and daidzein. All compounds are dissolved in 1 μl dimethyl sulfoxide (DMSO), and all cells including negative and positive (stimulation with KS-E) control cells were treated with 1 μl DMSO. (**C**) Mono Mac 6 cells were stimulated with hot water extracts prepared from whole beans as indicated in the Figure at 20-fold dilution, with or without AP-CF at the concentration of 100 μg/ml β-glucan. After 2 hours, the cells were harvested, and the THBS1 mRNA expressions were monitored using the real-time RT-PCR method. The data are represented as relative values compared with the mRNA expression in unstimulated control cells after normalization with GAPDH mRNA expression. Error bars indicate standard deviations calculated by three independent experiments.

The isoflavones, saponin, and lecithin are soybean components where beneficial effects on health have been reported^10–16^. In addition to these components, the THBS1 induction activity of lunasin, a peptide derived from soybeans has been reported^32^. Lunasin, a peptide originally found in soybeans, has been shown to display antitumor effects through the inhibition of histone acetylation^33^. To determine the soybean component which is involved in the induction of THBS1, the expression of THBS1 mRNA in Mono Mac 6 cells after stimulation with purified products of these various components was investigated. The results show that the expression of THBS1 mRNA is significantly increased after stimulation with an isoflavone mixture isolated from soybeans (Fig. 3A), and THBS1 mRNA is weakly induced after stimulation with saponin. The involvement of lunasin in the induction of THBS1 in non-tumorigenic human prostate epithelial cells has been reported^32^, in the Mono Mac 6 cells the THBS1 expression is however not affected by stimulation with lunasin. These results suggest that isoflavones are the main components involved in the induction of THBS1.

The isoflavones used in this study were mixtures of isoflavones isolated from soybeans and included daidzein, glycitein, and genistein which are known to be the major components of isoflavones in soybeans. To establish which isoflavone is involved in the induction of THBS1, the induction activities of these isoflavones were monitored using purified genistein, glycitein, and daidzein. The results indicate that the THBS1 mRNA expression is significantly increased after stimulation with all of these three isoflavones (Fig. 3B). The THBS1 mRNA induction activity of glycitein is weaker than daidzein and genistein, and the induced activities of daidzein and genistein are at similar levels. These results suggest that genistein and daidzein are the main components involved in the THBS1 induction after stimulation with KS-E.

Isoflavones are contained in a number of bean varieties, the seeds of Fabaceae family plants^34^. To investigate whether the THBS1 induction also takes place with other Fabaceae family beans, a conventional soybean (*Glycine max* (L.) Merrill), the Adzuki bean (*Vigna angularis* (Willd.) Ohwi & H. Ohashi), and a cultivar of the white-colored runner bean, Shirohanamame (*Phaseolus coccineus* cv. Shirohanamame) were tested, and the THBS1 induction activities of hot water extracts prepared from these beans were measured. As shown in Fig. 3C, the THBS1 mRNA expression is significantly increased after stimulation with the hot water extracts of these Fabaceae family beans.

### The kinases activated downstream of β-glucan receptors are involved in the enhancement of the THBS1 induction

**Figure 4:**
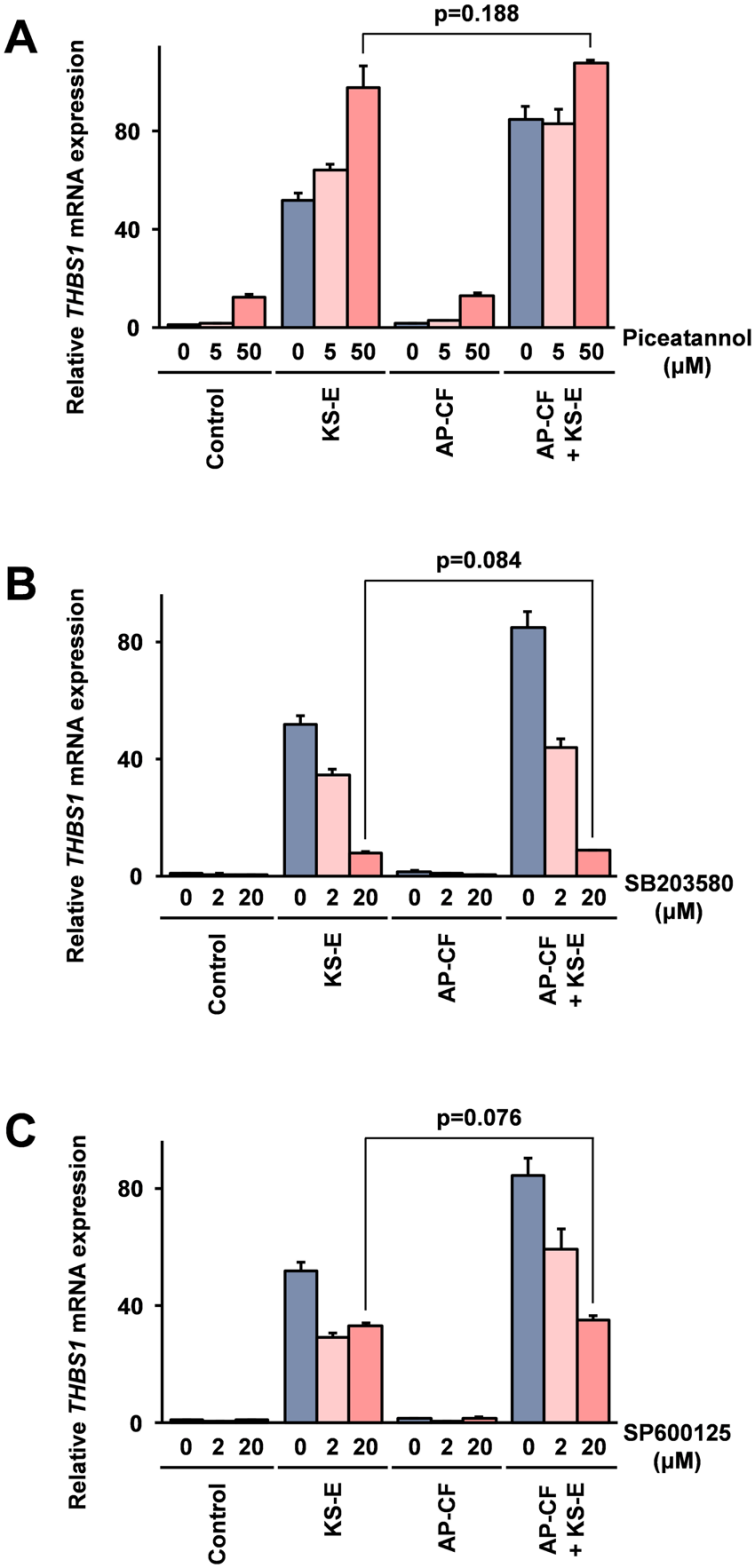
The Syk and its downstream kinases are involved in the enhancement of the THBS1 induction after stimulation with KS-E. Mono Mac 6 cells were treated for 1 hour with piceatannol (**A**), SB203580 (**B**), or SP600125 (**C**) at the concentrations indicated in the figure. Subsequently, the cells were stimulated with a 20-fold dilution of KS-E and AP-CF containing 100 μg/ml of β-glucan. After an additional 2-hour incubation period the cells were harvested, and the expressions of THBS1 mRNA in the cells were monitored using the real time RT-PCR method. The data are represented as relative values compared with the mRNA expression in the control cells after normalization with GAPDH mRNA expression. Error bars indicate standard deviations calculated by three independent experiments.

Spleen tyrosine kinase (Syk) is known as a kinase which is activated downstream of the β-glucan receptors CR3 and dectin-1^35, 36^. The p38 mitogen-activated protein kinase (p38 MAPK) and c-Jun N-terminal kinase (JNK) are known to be kinases which are activated downstream of the Syk-mediated signaling pathway^37^, and are involved in the cellular response to stimulation with β-glucan. To assess the involvement of these kinases in the induction of THBS1 after stimulation with KS-E, and the effect on the enhancement of the induction after co-stimulation with AP-CF, the effects of selective kinase inhibitors on the THBS1 mRNA induction were investigated. The results show that although the basal expression of THBS1 mRNA was increased after the treatment, the stimulation with KS-E induced THBS1 mRNA expression was not affected after the treatment with a Syk-selective inhibitor, piceatannol (Fig. 4A). However, the effect of co-stimulation with AP-CF on the enhancement of KS-E induced THBS1 mRNA expression was strongly inhibited by the piceatannol treatment. These results suggest that the Syk-mediated signaling pathway which is activated by β-glucan receptors is closely related in the AP-CF mediated enhancement of THBS1 induction after stimulation with KS-E.

Treatment with a selective inhibitor for p38 MAPK, SB203580, strongly inhibited THBS1 induction, while treatment with a selective inhibitor for JNK, SP600125 partially inhibited the induction of THBS1 mRNA expression after stimulation with KS-E (Fig. 4B and C). The enhancement of the THBS1 mRNA expression after co-stimulation with AP-CF was strongly inhibited after treatment with both the selective inhibitors for p38 MAPK (SB203580) and JNK (SP600125). These results suggest that signaling molecules which are known to be activated downstream of β-glucan receptors are involved in the enhancement of THBS1 induction after stimulation with KS-E.

## Discussion

The results of this study demonstrate that THBS1 is induced in a human monocyte derived cell line, Mono Mac 6 cells, after stimulation with KS-E, and this THBS1 induction is enhanced after co-stimulation with β-glucans including the β-glucan produced by the fermentation of *A*. *pullulans*. Several molecules have been reported as involved in recognition of β-glucans: CR3 (complement receptor 3)^38^, dectin-1^39^, langerin^40^, and lactosylceramide^41^. The data reported here using purified β-glucans demonstrates that the KS-E induced THBS1 expression is enhanced after co-stimulation with the purified β-glucans isolated from AP-CF, seaweed (laminarin), and *Trametes versicolor* (Krestin) (Fig. 2B). These β-glucans are structurally distinct^2, 30, 31^, and in particular laminarin is known to be an antagonist of dectin-1 that competitively inhibits activation of the dectin-1-mediated signaling pathway after stimulation with other β-glucans^42, 43^. Therefore, the results here may suggest that the effects of β-glucan on the enhancement of THBS1 induction after stimulation with KS-E are involved in the dectin-1-independent signaling pathway.

The analysis using components isolated from soybeans indicates that the isoflavones contained in soybeans are involved in the THBS1 induction activity of KS-E (Fig. 3A). The THBS1 induction activity of isoflavone mixtures is clearly lower than that of KS-E, a crude extract of Kurosengoku soybeans (Fig. 3A). In addition, single compounds of soybean isoflavones: genistein, glycitein, and daidzein, show weaker THBS1 induction activities than that of the isoflavone mixture (Fig. 3B). The isoflavone mixture used in this study was extracted and purified from soybeans, while the genistein, glycitein, and daidzein were chemically synthesized products. The reason why the THBS1 induction activities of the purified components are weaker than that of the less fully described ones is not fully elucidated, however the observations here may suggest that other unidentified compound(s) contained in soybeans are involved in the induction of THBS1.

As THBS1 is known to be a protein exhibiting anti-angiogenic activity^26, 27^, and anti-angiogenic activity has been established in the soybean isoflavone, genistein^44, 45^. In addition, anti-angiogenic activity of soy saponins has also been reported^46^. As shown in Fig. 3A, saponin isolated from soybeans also indicated THBS1 induction activity although the activity was weaker than that of the isoflavones. Overall the results here indicating that the stimulation with KS-E induces THBS1 in Mono Mac 6 cells, could in part be explained to involve the mechanism of the anti-angiogenic activity of soybeans.

The results using specific kinase inhibitors indicate that Syk, the tyrosine kinase activated downstream of dectin-1 and the CR3 receptor^35, 36^, is involved in the AP-CF-mediated enhancement activity of the KS-E induced THBS1 expression (Fig. 4A). However, the Syk-mediated signaling pathway is not involved in the induction of THBS1 after stimulation with KS-E. These results support the hypothesis that a β-glucan receptor-mediated pathway would be involved in the enhancement of KS-E induced in the THBS1 expression. Further, these results also suggest that activation of a different pathway from the β-glucan receptor-mediated signaling pathway is involved in the KS-E mediated THBS1 induction. The results using specific inhibitors for p38 MAPK, SB203580, and for JNK, SP600125 indicate that the KS-E induced THBS1 mRNA expression is mainly dependent on p38 MAPK, and both kinases are involved in the enhancement of the KS-E induced THBS1 mRNA expression (Fig. 4B and C). JNK is known to be a stress induced kinase activated by various stress stimuli including cycloheximide treatment^28^. This may explain the reason why the KS-E induced THBS1 mRNA expression was strongly enhanced after treatment with cycloheximide (Fig. 1C).

Many studies report that there are health beneficial effects of β-glucans and soybeans, and that several of these beneficial effects of β-glucan and soybeans overlap, including anti-tumor^3–5, 13, 14^ and anti-atherosclerotic^9, 47^ effects. On the other hand, combinational effects of treatment with β-glucan together with soybeans have not been fully assessed, and the report here would be the first to report cooperative effects of β-glucan and soybeans on cells. However, whether the cooperative effects of AP-CF and Kurosengoku soybeans shown in this study are actually effective *in vivo* is unknown. Further investigation is required to understand and elucidate the significance of the effects of Kurosengoku soybeans and β-glucan produced by *A*. *pullulans* on the health of organisms (humans).

## Methods

### Cell cultures

Mono Mac 6 cells (DSMZ ACC 124)^25^, a human monocyte-derived cell line exhibiting a mature monocyte phenotype, were cultured in RPMI1640 medium supplemented with 10% fetal bovine serum, 100 U/ml penicillin, 100 mg/ml streptomycin, 1% non-essential amino acids (Life Technologies, Carlsbad, CA, USA), and 1% OPI media supplement (Sigma-Aldrich, St. Louis, MO, USA). THP-1 cells (ATCC TIB-202)^29^, another human monocyte-derived cell line, were grown and maintained in RPMI 1640 medium supplemented with 10% fetal bovine serum, 100 U/ml penicillin, 100 mg/ml streptomycin. These cells were grown at 37 °C in 5% CO2 in a humidified incubator.

### Preparation of the hot water soluble extracts from various bean species

The Kurosengoku soybeans **(** *Glycine max* cv. Kurosengoku) used in this study were obtained from Mr. Yukio Takada, board chairman, the Kurosengoku business cooperative association, Hokuryu town, Hokkaido, Japan. Conventional soybeans, *Glycine max* (L.) Merrill, Adzuki beans, *Vigna angularis* (Willd.) Ohwi & H. Ohashi, and a cultivar of the white-colored runner bean, Shirohanamame (*Phaseolus coccineus* cv. Shirohanamame) were purchased as commercially available products. The black soybeans, Kurosengoku, were ground at 16 °C. A 10% (w/v) suspension of Kurosengoku powder in distilled water was autoclaved at 120 °C for 30 min. After lipids and insoluble debris were removed using filter paper, the water soluble extracts of Kurosengoku were sterilized by further autoclaving, and used in this study. To prepare a hot water extract from whole beans, a mixture of 10% (w/v) of whole beans was placed in water, autoclaved at 120 °C for 30 min, and the supernatant was used for the study.

### Preparation of *Aureobasidium pullulans* cultured fluid (AP-CF) containing β-glucan

The AP-CF was prepared as described elsewhere^6^. The AP-CF made with Kurosengoku soybeans (kAP-CF) was prepared in a manner similar to that of conventional AP-CF using Kurosengoku soybeans instead of rice bran as the nitrogen source. Briefly, *A*. *pullulans* was grown at 24.5 °C for 4 days, in a medium containing 2% Sucrose, 0.3% powdered Kurosengoku soybeans, 0.08% sodium L-ascorbate, and 0.02% L-ascorbic acid. This cultured medium was heated at 90 °C for 30 min and this heat-sterilized cultured medium was used for the study. The concentration of β-glucan in the kAP-CF was estimated to be 6 mg/ml. The purified β-glucan produced by *A*. *pullulans* (AP-PG) was prepared using ultrafiltration with a cut-off molecular weight of 20,000 followed by ethanol precipitation as previously described^6^. A protein conjugated β-glucan derived from *Trametes versicolor* CM-101, Krestin (Kureha Chemical Industry, Tokyo, Japan)^31^ and a *Laminaria digitata*-derived β-glucan, laminarin (Sigma-Aldrich)^30^ were purchased as commercially available products.

### Real-time reverse transcription polymerase chain reaction (RT-PCR)

To monitor the Thrombospondin-1 (THBS1) mRNA expression, the total RNA was extracted from cultured cells using TRIzol reagent (Invitrogen, Carlsbad, CA, USA). The isolated total RNAs were treated with DNaseI (Takara, Shiga, Japan) and then subjected to oligo-dT- and random-primed reverse transcription using ReverTra Ace (Toyobo, Osaka, Japan). Real-time RT-PCR was performed using SYBR Premix Ex Taq II (Takara). The PCR reactions and analysis of mRNA expressions were performed using the CFX96 Real-Time PCR Detection System (Bio-Rad, Hercules, CA, USA). Each procedure was performed according to the manufacturer’s protocol. The following specific primer set for THBS1 was used in this study: sense primer: 5′-ATGGAGAATGCTGTCCTCGC-3′, antisense primer: 5′-CCATTGCCACAGCTCGTAGA-3′.

### Enzyme-linked immunosorbent assay (ELISA)

The concentration of THBS1 in the supernatant of the culture medium was quantified using a commercially available ELISA kit (Quantikine ELISA human Thrombospondin-1, R&D Systems, Minneapolis, MN, USA) in accordance with the manufacturer’s protocol.

### Assays using selective kinase inhibitors

Selective protein kinase inhibitors, piceatannol, SB203580, and SP600125 were purchased as commercially available products (Abcam, Cambridge, MA, USA). Piceatannol (50 mM), and SB203580 (20 mM) and SP600125 (20 mM) were dissolved in dimethyl sulfoxide (DMSO) as stock solutions. The Mono Mac 6 cells were treated with piaceatannol, SB203580, or SP600125 for 1 hour. The concentrations of DMSO in the culture medium were equalized to 0.1%. After the treatment, the cells were stimulated with a 20-fold dilution of the hot water extracts of Kurosengoku soybeans (KS-E) and AP-CF at the concentration of 100 μg/ml β-glucan.

### Components isolated from soybeans

Isoflavone mixture isolated from soybeans (Isoflavone Aglycone Mixture B [Genistein ≥50%], from Soybean), and purified isoflavones (daidzein, glycitein, and genistein) were purchased from Nagara Science, Gifu, Japan. Soybean derived saponin and lecithin were obtained commercially (Wako Pure Chemical, Osaka, Japan).

### Statistical analysis

To determine statistically significant differences between pairs of data, a two-tailed unpaired Student’s t-test was performed in this study. A value of p < 0.05 was used to show statistical significance.
